# A Functional Ser326Cys Polymorphism in *hOGG1* Is Associated with Noise-Induced Hearing Loss in a Chinese Population

**DOI:** 10.1371/journal.pone.0089662

**Published:** 2014-03-05

**Authors:** Huanxi Shen, Jinglian Cao, Zhiqiang Hong, Kai Liu, Jian Shi, Lu Ding, Hengdong Zhang, Cheng Du, Qian Li, Zhengdong Zhang, Baoli Zhu

**Affiliations:** 1 Kunshan Municipal Center for Disease Prevention and Control, Kunshan, China; 2 Department of Environmental Genomics, Jiangsu Key Laboratory of Cancer Biomarkers, Prevention and Treatment, Cancer Center, Nanjing Medical University, Nanjing, China; 3 Institute of Occupational Disease Prevention, Jiangsu Provincial Center for Disease Prevention and Control, Nanjing, China; 4 Department of Disease Prevention and Control of Yizheng Hospital, Drum Tower Hospital Group of Nanjing, Yizheng, China; 5 Department of Genetic Toxicology, the Key Laboratory of Modern Toxicology of Ministry of Education, School of Public Health, Nanjing Medical University, Nanjing, China; 6 The First People's Hospital of Kunshan, Kunshan, China; Institute of Molecular Genetics IMG-CNR, Italy

## Abstract

DNA damage to cochlear hair cells caused by 8-oxoguanine (8-oxoG) is essential for the development of noise-induced hearing loss (NIHL). Human 8-oxoG DNA glycosylase1 (hOGG1) is a key enzyme in the base excision repair (BER) pathway that eliminates 8-oxoG. Many epidemiological and functional studies have suggested that the *hOGG1* Ser326Cys polymorphism (rs1052133) is associated with many diseases. The purpose of this investigation was to investigate whether the *hOGG1* Ser326Cys polymorphism in the human BER pathway is associated with genetic susceptibility to NIHL in a Chinese population. This polymorphism was genotyped among 612 workers with NIHL and 615 workers with normal hearing. We found that individuals with the *hOGG1* Cys/Cys genotype had a statistically significantly increased risk of NIHL compared with those who carried the *hOGG1* Ser/Ser genotype (adjusted OR = 1.59, 95% CI = 1.13–2.25) and this increased risk was more pronounced among the workers in the 15- to 25- and >25-year noise exposure time, 85–92 dB(A) noise exposure level, ever smoking, and ever drinking groups, similar effects were also observed in a recessive model. In summary, our data suggested that the *hOGG1* Cys/Cys genotype may be a genetic susceptibility marker for NIHL in the Chinese Han population.

## Introduction

Noise-induced hearing loss (NIHL) is a common sensorineural impairment that is often caused by continuous and regular exposure to noise. NIHL is a leading occupational health risk in industrialised countries worldwide and the second most common form of sensorineural hearing impairment [1]. In the United States, 10 million people have noise-related hearing loss (http://www.cdc.gov/NIOSH/), and in China, the number of workers with NIHL has increased 77.8% in the last three years (2010–2012, http://www.aqsc.cn).

NIHL is a complex disease that is induced by a combination of environmental and genetic factors. It is well known that noise, chemicals such as organic solvents, ototoxic substances (e.g., aminoglycosides), heat, vibrations, smoking, and medical factors (increased blood pressure, cholesterol, and reduced iris pigmentation) are risk factors for NIHL [2], [3]. To identify susceptible workers and to develop new therapies that prevent NIHL, increasing new research is focused on an understanding of NIHL at the molecular level.

In the last 20 years, several animal studies have demonstrated that knockout mice, including *SOD −/−* [4], *GPX −/−* [5], *PMCA2 −/−* [6], and *CDH23 +/−* [7], have shown more sensitivity to noise than their wild-type littermates. These studies have suggested that deficits in genes that disrupt different pathways (e.g., oxidative stress and potassium recycling) and critical structures of the cochlea (e.g., stereocilia) contribute to an increase in the susceptibility of the inner ear to noise [1]. However, in humans, the discovery of susceptibility genes to NIHL has encountered many difficulties; no formal heritability studies have been performed to date. Furthermore, it is difficult to collect data from families or twins in which all subjects have been exposed to similar noise environments. However, association studies adopting a candidate gene approach have opened the door to discovering NIHL susceptibility genes [1]. Over the last 10 years, several studies have found that potassium-recycling pathway genes [8], [9], monogenic deafness genes [10], [11], Hsp70 genes [11], [12], and oxidative stress genes [13], [14], [15], [16], [17] have been associated with NIHL.

DNA damage to cochlear hair cells is essential for the development of NIHL, especially in the context of exposure to high noise levels [18]. The end result is cochlear hair death from a combination of necrosis and apoptosis. Correspondingly, several DNA repair pathways, including base excision repair (BER) [19], [20], [21], mismatch repair (MMR), nucleotide excision repair (NER), and DNA double-strain break repair [19], have evolved to maintain the genetic integrity of these cells and to prevent cell death. *Totonchy MB et al.* [22] also reported that DNA repair was important in the maintenance of hearing.

The 8-oxoguanine (8-oxoG) DNA lesion, caused by reactive oxygen species (ROS), is one of the most common forms of oxidative damage to DNA and leads to G: C to T: A transversions, causing carcinogenesis [23]. Human 8-oxoG DNA glycosylase1 (hOGG1) is a key enzyme in the BER pathway that eliminates 8-oxoG [19], [24].

Many epidemiological and functional studies have suggested that the Ser326Cys polymorphism (rs1052133) in exon 7 of *hOGG1* gene may affect the activity of hOGG1 enzyme [25], [26] and may therefore serve as a genetic marker for susceptibility to many diseases [27], [28], [29], [30]; however, thus far, there have been no studies of association between the *hOGG1* Ser326Cys polymorphism and risk of NIHL.

To determine whether the *hOGG1* Ser326Cys polymorphism in the human BER pathway was associated with susceptibility to NIHL in the Chinese population, we genotyped 615 NIHL workers and 615 normal hearing workers and compared the genotype frequencies between these two groups.

## Materials and Methods

### 2.1 Subjects

This study included 615 NIHL workers and 615 normal hearing workers from the Datun Coal and Electricity Company (Xuzhou, China. 32 NIHL workers and 32 normal hearing workers were selected), Chenguang Machinery Manufacturing Group Corporation (Nanjing, China. 138 NIHL workers and 138 normal hearing workers were selected), and Yizheng Chemical Fiber Company Limited (Yizheng, China. 445 NIHL workers and 445 normal hearing workers were selected) between April 2010 and May 2011. All subjects (age range, 21–59 years) were Han Chinese. The detail population of information has been described in our previous study [16]. Briefly, these subjects had been continuously employed in these plants for at least one year, and all of them had their hearing tested once a year according to the Technical Specifications for Occupational Health Surveillance (2002) of China. We enrolled volunteers when workers exposed to occupational noise had their health examination; the inclusion criteria we used have been described in detail previously. In general, the selected workers had not been exposed commonly to chemical or physical factors associated with hearing loss (e.g., heat, vibrations, and organic solvents), had no record of military service, and had no medical factors or diseases that could affect hearing. The workers we selected did not regularly use hearing protection. The noise exposure time for each work was recorded according to their occupational health surveillance files which contained their exact noise exposure time. The subjects were recorded as ever smokers if they had smoked 100 cigarettes or more in their lifetimes. The subjects were recorded as ever drinkers if they had consumed three or more alcoholic drinks per week for at least one year [16]. This study protocol was approved by the institutional review board of Nanjing Medical University, and written informed consent was obtained from all participants.

### 2.2 Questionnaire

The structured questionnaires were administered in face-to-face interviews conducted by our topic-based group for each subject. The information requested in the questionnaire included informed consent, demographic characteristics, lifestyle habits (smoking and alcohol consumption), history of ototoxic drug use, work history, noise exposure, physical and chemical factor exposure, hearing protection use, disease history, family history of deafness, and so on. All of the workers had been interviewed, and 1,230 useable questionnaires were collected.

### 2.3 Audiological status assessment and environmental noise measurement

As we have described in a previous study [16], an otolaryngologist conducted 500, 1,000, 2,000, 3,000, 4,000, and 6,000 pure-tone air hearing threshold tests in a sound-attenuating chamber with a background noise level of less than 25 dB(A). The workers were required to avoid a noisy environment for 12–48 h before they were tested. According to the Diagnostic Criteria of Occupational Noise-Induced Hearing Loss (Chinese Occupational Health Standard, GBZ49-2002, http://www.zybw.net) and the Technical Specifications for Occupational Health Surveillance, an ascending method in 5 dB(A) steps was adopted to ascertain the hearing threshold levels of both ears. The final threshold value for each ear was defined as the lowest signal intensity that a subject detected with a minimum of 3 trials. Hearing thresholds of less than 25 dB in both high- and low-frequency ranges were defined as normal. Correspondingly, hearing thresholds worse than 25 dB in the high-frequency range or in both high- and low-frequency ranges were defined as NIHL. However, in our study a high-frequency hearing threshold worse than 25 dB was recorded only because NIHL workers (35 workers) with both low- and high- frequency hearing worse than 25 dB were required to be removed from noisy environments according to the Diagnostic Criteria of Occupational Noise-Induced Hearing Loss (GBZ49-2002).

We used individual sound pressure noise meters (Noise-Pro, Quest, Oconomowoc WI, USA) to detect noise exposure levels in the workplace; they were worn by 1 to 10 workers in each workplace during the work shift, three times a year, as mandated by the China National Criteria for Noise in the Workplace. Additionally, the Noise-Pro was used at 10 AM, 3 PM, and 5 PM for three consecutive days at each workplace to test the noise exposure levels.

In the final analysis, the normal hearing workers (615 workers) were matched with the NIHL workers (615 workers) by age, sex, and similarity of occupational exposures (including noise exposure time, noise exposure level and so on). All subjects donated 2 ml venous blood samples for DNA extraction.

### 2.4 Genotyping of hOGG1 Ser326Cys polymorphisms

Genomic DNA was isolated from the peripheral blood samples according to standard procedures using a TianGen DNA extraction kit (Beijing, China). The *hOGG1* Ser326Cys polymorphism was detected using the TaqMan SNP Genotyping assay and the 96-well ABI 7900HT Real Time PCR System (Applied Bio-systems, Foster City, CA, USA). The primer sequences were 5′-CCTCCTACAGGTGCTGTTCAGTG-3′ and 5′-ACCCTTTCTGCGCTT- TGCT-3′, and the probes were 5′-FAM- CCAATGCCGCCATG-MGB-3′ and 5′-HEX-CGCCAATCCCGCCA-MGB-3′, which were designed and manufactured by Applied Biosystems Inc. (Foster City, CA, USA). The final volume for each reaction was 10 µl, consisting of 2.5 µl ddH_2_O, 5 µl TaqMan Master Mix, 0.5 µl F-prime, 0.5 µl R-prime, 0.25 µl FAM-probe, 0.25 µl HEX-probe (Applied Biosystems Inc.), and 10 ng DNA. The PCR profile consisted of an initial denaturation step at 50°C for 2 min, 95°C for 10 min, and 40 cycles of 95°C for 15 s and 60°C for 1 min. The fluorescence level was detected using an ABI 7900HT Real-Time PCR System. The allele frequencies were determined using ABI SDS 2.3 software.

The genotypes of 3 NIHL workers could not be determined, although 3 attempts were made. Therefore, 612 NIHL workers and 615 normal hearing workers were analysed in total. Two persons independently performed the genotyping in a blinded fashion. More than 10% of the samples were randomly selected for confirmation, and the reproducibility was 100%.

### 2.5 Statistical analysis

All of the data were reorganised and entered with EpiData 3.1, and the statistical analysis was performed using SAS statistical software (version 9.1.3; SAS Institute, Cary, NC, USA).

Continuous data were analysed by independent-sample two-sided t tests. Categorical data were computed by two-sided χ^2^ tests. Hardy-Weinberg equilibrium was tested by a goodness-of-fit χ^2^ test. Multivariate logistic regressions were used to compute odds ratios (ORs) and 95% confidence intervals (95% CIs) to test the associations of different genotypes with noise susceptibility. Adjusted ORs and 95% CIs were computed by multivariate logistic regression adjusted for age, sex, smoking status, and drinking status. Furthermore, the stratification analyses were performed according to the subsections of noise exposure time, noise exposure level, smoking status, and drinking status, to estimate the different combinations of *hOGG1* genetic variants between NIHL workers and normal hearing workers. Multivariate logistic regressions were also used to analyze gene-environment interactions. All of the tests were two-sided, and a *P*-value <0.05 was considered statistically significant.

## Results

### 3.1 Subject characteristics

The demographic and occupational characteristics of 612 NIHL workers and 615 normal hearing workers are shown in Table 1. Overall, there were no significant differences in the distribution of age, sex, smoking status, drinking status, exposure level, or exposure time between the NIHL and normal hearing workers. We did observe that the average threshold value of the NIHL workers was over two times greater than that of the normal hearing workers [37.2 dB(A) *vs.* 14.1 dB(A)]. The majority of the smokers and drinkers in our cohort were light smokers and light drinkers (the average was 4.8 cigarettes per day and 8.5 g of alcohol per day separately); we did not analyse the effects of smoking and drinking quantitatively.

**Table 1:** Demographic and occupational characteristics of the NIHL and normal hearing workers.

Variables	NIHL workers (n = 612)		Normal hearing workers (n = 615)		*P* a
	N	%	N	%	
Age (years)	40.4±6.3		40.4±5.9		0.979^b^
<35	122	19.9	120	19.5	0.963
35–45	368	60.2	369	60.0	
>45	122	19.9	126	20.5	
Sex					
Male	565	92.3	564	91.7	0.692
Female	47	7.7	51	8.3	
Smoking status					
Never	366	59.8	359	58.4	0.611
Ever	246	40.2	256	41.6	
Drinking status					
Never	259	42.3	256	41.6	0.805
Ever	353	57.7	359	58.4	
Exposure level [dB(A)]	87.1±7.7		87.0±7.6		0.804^b^
<85	234	38.2	246	40.0	0.735
85–92	161	26.3	151	24.5	
>92	217	35.5	218	35.5	
Exposure time (years)	18.6±7.6		18.2±7.4		0.403^b^
<15	189	30.9	194	31.5	0.645
15–25	308	50.3	318	51.7	
>25	115	18.8	103	16.8	
Threshold [dB]	37.2±11.9		14.1±4.1		**<0.001** b

a Two-sided χ^2^ test was used for comparing the frequency distribution.

b Two-sided *t*-test was used for comparing the mean values of the continuous variables.

### 3.2 Associations of the hOGG1 polymorphisms with the susceptibility to NIHL

The observed genotypes and allele frequencies of *hOGG1* among the NIHL workers and normal hearing workers and their associations with the risk of NIHL are presented in Table 2. The allele frequency of *hOGG1* Ser326Cys among the normal hearing workers was consistent with Hardy–Weinberg equilibrium (*P* = 0.223). The frequency of the *hOGG1* Cys/Cys genotype in the normal hearing workers (12.7%), which is similar to that reported in a previous study in a Chinese population (the frequency of *hOGG1* Cys/Cys genotype is 13.4%) [31], was statistically lower than the frequency in the NIHL workers (17.9%, *P* = 0.008). Multivariate logistic regression analyses also revealed that individuals with the *hOGG1* Cys/Cys genotype had a 1.59-fold risk of NIHL compared with individuals carrying a Ser/Ser genotype (adjusted OR = 1.59, 95% CI = 1.13–2.25). When we combined the Ser/Ser and Ser/Cys genotypes as a reference to conduct a recessive model, the number of workers with the Cys/Cys genotype was greater among the NIHL group than in the normal hearing worker group (17.9% *vs.* 12.7%, *P* = 0.010); workers who carried the Cys/Cys genotype had a higher risk of NIHL (1.52, 1.11–2.08). We also found that there were more Cys allele individuals among the NIHL workers than among the normal hearing workers (41.9% *vs.* 37.2%, *P* = 0.017). The multivariate logistic regression analysis revealed that the workers with the Cys allele had a higher risk of NIHL than the normal hearing workers (OR = 1.22, 95% CI = 1.04–1.43).

**Table 2:** Association of the *hOGG1* Ser326Cys polymorphism with the risk of NIHL.

Genotypes	NIHL workers (n = 612)		Normal hearing workers (n = 615)		OR (95% CI)	Adjusted^a^ OR (95% CI)	*P* b
	N	%	N	%			
Ser/Ser	209	34.2	236	38.4	1.00 (reference)	1.00 (reference)	
Ser/Cys	293	47.9	301	48.9	1.10 (0.86–1.41)	1.09 (0.86–1.40)	0.451
Cys/Cys	110	17.9	78	12.7	**1.59 (1.13–2.25)**	**1.59 (1.13–2.25)**	**0.008**
Ser/Ser+Ser/Cys	502	82.1	537	87.3	1.00 (reference)	1.00 (reference)	
Cys/Cys	110	17.9	78	12.7	**1.51 (1.10–2.07)**	**1.52 (1.11–2.08)**	**0.010**
Ser allele	711	58.1	773	62.8	1.00		
Cys allele	513	41.9	457	37.2	**1.22 (1.04–1.43)**		**0.017**

a Adjusted for age, sex, smoking, and drinking status.

b Two-sided χ^2^ test for the frequency distributions of selected variables between the hearing loss workers and normal hearing workers.

### 3.3 Stratification analysis between the hOGG1 polymorphism and risk of NIHL

The results of the stratification analysis are presented in Tables 3 and Table 4. In Table 3, we found that the individuals with the *hOGG1* Cys/Cys genotype were more susceptible to NIHL than those who carrying the *hOGG1* Ser/Ser genotype, both in the 15- to 25-year noise exposure group (adjusted OR = 1.67, 95% CI = 1.02–2.72) and in the >25-year noise exposure group (2.71, 1.16–6.36). Similar effects were also noted for smoking and drinking status; ever smoking or ever drinking subjects with the *hOGG1* Cys/Cys genotype were more susceptible to NIHL. But in the stratification of noise exposure level, we only found that the *hOGG1* Cys/Cys genotype carriers with 85 to 92 dB(A) exposure levels were at a significantly increased risk of NIHL (3.34, 1.59–7.02); no effects were observed in either the <85 dB(A) or >92 dB(A) noise-exposure levels.

**Table 3:** Stratified analysis of the *hOGG1* Ser326Cys polymorphism (Cys/Cys *vs.* Ser/Ser genotype) associated with NIHL risk.

Variables	NIHL workers		Normal hearing workers		OR (95% CI)	Adjusted OR (95% CI)^a^	*P* b
	Ser/Ser (n = 209)	Cys/Cys (n = 110)	Ser/Ser (n = 236)	Cys/Cys (n = 78)			
	N (%)	N (%)	N (%)	N (%)			
Exposure time (years)							0.130^c^
<15	69 (36.3)	27 (14.2)	69 (36.3)	25 (14.2)	1.08 (0.57–2.04)	1.09 (0.57–2.07)	0.813
15–25	103 (31.7)	53 (16.3)	129 (39.7)	40 (12.3)	**1.66 (1.02–2.70)**	**1.67 (1.02–2.72)**	**0.040**
>25	37 (31.4)	30 (25.4)	38 (32.2)	13 (11.0)	**2.37 (1.07–5.24)**	**2.71 (1.16–6.36)**	**0.031**
Exposure level [dB(A)]							0.363^c^
<85	84 (31.9)	47 (17.9)	97 (36.9)	35 (13.3)	1.55 (0.92–2.62)	1.53 (0.89–2.62)	0.101
85–92	51 (30.7)	36 (21.7)	64 (38.6)	15 (9.0)	**3.01 (1.49–6.10)**	**3.34 (1.59–7.02)**	**0.002**
>92	74 (36.3)	27 (13.2)	75 (36.8)	28 (13.7)	0.98 (0.53–1.81)	0.98 (0.53–1.82)	0.942
Smoking status							0.731^c^
Never	128 (34.2)	60 (16.0)	142 (38.0)	44 (11.8)	1.51 (0.96–2.39)	1.51 (0.96–2.39)	0.075
Ever	81 (31.3)	50 (19.3)	94 (36.3)	34 (13.1)	**1.71 (1.01–2.89)**	**1.73 (1.02–2.95)**	**0.046**
Drinking status							0.318^c^
Never	88 (34.0)	40 (15.4)	97 (37.5)	34 (13.1)	1.30 (0.76–2.23)	1.33 (0.77–2.29)	0.346
Ever	121 (32.3)	70 (18.7)	139 (37.2)	44 (11.8)	**1.83 (1.17–2.86)**	**1.82 (1.16–2.86)**	**0.008**

a Adjusted for age, sex, smoking, and drinking.

b Two-sided χ^2^ test for the frequency distributions of the selected variables between the NIHL workers and normal hearing workers.

c Gene-environment interaction *P* values for the *hOGG1* Ser326Cys polymorphism and NIHL risk factors.

**Table 4:** Stratified analysis of the *hOGG1* Ser326Cys polymorphism (Cys/Cys *vs.* Ser/Ser+Ser/Cys genotype) associated with NIHL risk.

Variables	NIHL workers		Normal hearing workers		OR (95% CI)	Adjusted OR (95% CI)^a^	*P* b
	Ser/Ser+Ser/Cys (n = 502)	Cys/Cys (n = 110)	Ser/Ser+Ser/Cys (n = 537)	Cys/Cys (n = 78)			
	N (%)	N (%)	N (%)	N (%)			
Exposure time (years)							0.072^c^
<15	162 (42.3)	27 (7.1)	169 (44.1)	25 (6.5)	1.13 (0.63–2.02)	1.15 (0.64–2.08)	0.689
15–25	255 (40.7)	53 (8.5)	278 (44.4)	40 (6.4)	1.45 (0.93–2.25)	1.47 (0.94–2.29)	0.104
>25	85 (39.0)	30 (13.8)	90 (41.3)	13 (5.9)	**2.44 (1.20–4.99)**	**2.48 (1.20–5.14)**	**0.013**
Exposure level [dB(A)]							0.415^c^
<85	187 (39.0)	47 (9.8)	211 (44.0)	35 (7.2)	1.52 (0.94–2.45)	1.50 (0.93–2.43)	0.088
85–92	125 (40.1)	36 (11.5)	136 (43.6)	15 (4.8)	**2.61 (1.36–5.00)**	**2.83 (1.46–5.49)**	**0.003**
>92	190 (43.7)	27 (6.2)	190 (43.7)	28 (6.4)	0.96 (0.55–1.70)	0.95 (0.54–1.68)	0.900
Smoking status							0.593^c^
Never	306 (42.2)	60 (8.3)	315 (43.5)	44 (6.1)	1.40 (0.92–2.14)	1.42 (0.93–2.16)	0.112
Ever	196 (39.0)	50 (10.0)	222 (44.2)	34 (6.8)	**1.67 (1.04–2.68)**	**1.68 (1.04–2.70)**	**0.035**
Drinking status							0.231^c^
Never	219 (42.5)	40 (7.8)	222 (43.1)	34 (6.6)	1.19 (0.73–1.95)	1.21 (0.74–1.99)	0.484
Ever	283 (39.8)	70 (9.8)	315 (44.2)	44 (6.2)	**1.77 (1.18–2.67)**	**1.78 (1.18–2.68)**	**0.006**

a Adjusted for age, sex, smoking, and drinking.

b Two-sided χ^2^ test for the frequency distributions of selected variables between the NIHL workers and normal hearing workers.

c Gene-environment interaction *P* values for the *hOGG1* Ser326Cys polymorphism and NIHL risk factors.

A stratification analysis between the *hOGG1* recessive model and risk of NIHL is shown in Table 4. The subjects with the *hOGG1* Cys/Cys genotype had an increased risk of NIHL in the >25-year group (adjusted OR = 2.48, 95% CI = 1.20–5.14), the 85 to 92 dB(A) noise exposure level group (2.83, 1.46–5.49), the ever smoking group (1.68, 1.04–2.70), and the ever drinking group (1.78, 1.18–2.68).

Gene-environment interactions were also analyzed between the *hOGG1* Cys/Cys genotype and NIHL risk factors (noise exposure time, noise exposure level, smoking and drinking), but no gene-environment interactions were found in this study (*P*>0.05).

## Discussion

In this case-control study, we found that the *hOGG1* Cys/Cys genotype was statistically significantly associated with NIHL. Moreover, the synergistic effects of the *hOGG1* Ser326Cys polymorphism and noise exposure time, noise exposure level, smoking status, and drinking status on NIHL were observed in our study.

Several studies have reported that the *hOGG1* Ser326Cys polymorphism was associated with risk of cancers and various metabolic disorders [21], [29], [30], [32]. However, there have been no studies published exploring the *hOGG1* Ser326Cys polymorphism and NIHL risk among noise exposure subjects. To the best of our knowledge, this study is the first to investigate the association between the *hOGG1* Ser326Cys polymorphism and NIHL risk in a Chinese population.

Caused by ROS, 8-oxoG in DNA is a major form of oxidative damage, which may lead to mutagenesis or carcinogenesis [23] and which can be repaired by the BER pathway. The hOGG1 enzyme in the BER pathway, which is encoded by the *hOGG1* gene on chromosome 3p26, recognises the 8-oxoG lesions and catalyses the cleavage of the glycosidic bond between the modified base and sugar moiety, leaving an abasic, apurinic/apyrimidinic site (AP-site) in DNA, and the AP-endonuclease-1 (APEX/APE1) enzyme then acts upon it [19]. Multiple functional studies have revealed that the glycosylase activity of the Cys326 variant of the hOGG1 enzyme (Cys326-hOGG1 enzyme) is more sensitive to inactivation by oxidising agents compared with the Ser326-hOGG1 enzyme [25], [33]. The activity for the repair of 8-oxoG was approximately 7-fold greater in the hOGG1-Ser326 protein than in the hOGG1-Cys326 protein in a complementation assay of an *E. coli* mutant defective in the repair of 8-oxoG [34]. Our study also found there were more individuals with the *hOGG1* Cys/Cys genotype among NIHL workers than among normal hearing workers, suggesting that subjects carrying the *hOGG1* Cys/Cys genotype may have lower activity for repairing 8-oxoG damage, thereby increasing the risk of NIHL.

Notably, when the noise exposure time was combined with the *hOGG1* Cys/Cys genotype, the effects were more significant. NIHL is positively correlated with noise exposure time; when workers are exposed to noise for a longer time, the cumulative noise exposure (CNE) is greater, and they are more susceptible to NIHL [3]. Moreover, many workers with longer noise exposure times are older and may be influenced by age-related hearing impairment (ARHI). A previous study has also reported that there is a significantly age-associated decrease in DNA repair capacity [35].

Noise exposure levels are important for the activation of the antioxidant system. In chinchillas, *Jacono AA* [36] observed no increase in the antioxidant system after a conditioning noise exposure of 90 dB SPL (0.5 kHz, 6 h per day for 6 days), but a large increase in the antioxidant system in response to 105 dB SPL (0.5 kHz, 4 h). Positively induced by ROS, 8-oxoG is correlated with noise exposure level and also has a positive correlation with the expression of DNA repair enzymes [37]. NIHL is a progressive, cumulative disease; most workers in the >92 dB(A) noise exposure group were often transferred to a no-noise exposure position after working for a few years, as we described previously [16]. The subjects in the <85 dB(A) and >92 dB(A) groups had low cumulative noise exposures, while individuals in the 85 to 92 dB(A) group were assumed to have had sufficient cumulative noise exposure.

Smoking may accelerate NIHL; some studies have reported the adverse effects of smoking on hearing [38], [39]. However, a few studies have not identified any associations between smoking and hearing loss [40]. Our study showed that smoking was associated with NIHL. Workers with the *hOGG1* Cys/Cys genotype had a 1.68-fold increased risk of NIHL than workers who carried the *hOGG1* Ser/Ser genotype among ever smokers. The effect was less pronounced when all subjects were included in the analysis (adjusted OR = 1.59, less than 1.68). In the recessive model, a similar phenomenon was also observed. A possible explanation for the underlying pathogenic mechanism may be the well-known vascular changes and the consequent cochlear hypoxia related to both smoking and noise exposure. Adverse effects were also observed between drinking and NIHL in this study, which was consistent with previous studies [41], [42]. However, some studies have reported that drinking alcohol may not either influence hearing or be a protective factor [43], [44]; more study is required to confirm this possibility.

Compared with other studies, ours was the first to investigate the association between the functional *hOGG1* Ser326Cys polymorphism and NIHL risk. Moreover, thousands of workers who had been exposed to steady noise for up to 20 years or more but who were less exposed to other occupational hazards were enrolled in our study. Furthermore, all of our NIHL workers were in an early stage of NIHL according to GBZ49-2002; additionally, individual noise meters were used to assess the noise environment so that the results would reflect real exposure levels. One limitation of this study was that the NIHL workers with both low- and high- frequency hearing ranges worse than 25 dB were all transferred from noisy environments; therefore, selection bias may exist in our study.

In conclusion, our data suggested that the *hOGG1* Cys/Cys genotype may be a genetic susceptibility marker for NIHL in the Han Chinese population, especially in the 15- to 25-year and >25-year noise exposure time, 85 to 92 dB(A) noise exposure level, ever smoking, and ever drinking groups. To confirm our findings, further case-control or cohort studies with more subjects (enrolling all NIHL workers) and *in vivo* functional evaluations are needed in the future.
